# Fiber-Coupled Fully Integrated Spin-Exchange Relaxation-Free Atomic Magnetometer for Functional Biomagnetic Measurements

**DOI:** 10.3390/s26092593

**Published:** 2026-04-22

**Authors:** Wennuo Jiang, Jianjun Li, Xinkun Li, Yuanxing Liu

**Affiliations:** 1 College of Optical Science and Engineering, Zhejiang University, Hangzhou 310027, China; 2 Beijing Key Laboratory of Quantum Sensing and Precision Measurement, and Center for Quantum Information Technology, and Institute of Quantum Electronics, Peking University, Beijing 100871, China; 3 Laboratory of Solid-State Optoelectronic Information Technology, Institute of Semiconductors, Chinese Academy of Sciences, Beijing 100083, China; 4 Institute of Quantum Sensing and School of Physics, Zhejiang University, Hangzhou 310027, China; 5 Institute of Fundamental and Transdisciplinary Research, Zhejiang University, Hangzhou 310027, China

**Keywords:** spin-exchange relaxation-free, atomic magnetometer, fully integrated, biomagnetic measurements

## Abstract

The atomic magnetometer (AM), operating within the spin-exchange relaxation-free (SERF) regime, boasts numerous advantageous qualities, including ultrahigh sensitivity, exceptional spatial resolution, and minimal power consumption. Consequently, it emerges as a promising alternative to superconducting quantum interference devices in biomagnetic measurement applications. This paper details the development of a fully integrated SERF AM system comprising a compact sensor head and corresponding control electronics. Utilizing a 4 mm × 4 mm × 4 mm cubic vapor cell, we have successfully integrated the compact sensor into a 9 ${\mathrm{cm}}^{3}$ volume employing a single-beam scheme facilitated by a polarization-maintaining fiber. The in-house control electronics encompass essential components, such as the laser driver, coil driver, vapor-cell temperature controller, and transimpedance amplifier. As a result, the fully integrated SERF AM achieves a sensitivity of 25 $\mathrm{fT}/{\mathrm{Hz}}^{1/2}$@5∼100 Hz, accompanied by a bandwidth of 193 Hz, meeting the necessary criteria for magnetocardiography (MCG) and magnetoencephalography (MEG) measurements. Furthermore, the fully integrated SERF AM successfully records typical MCG and alpha rhythm MEG signals, showcasing immense potential for biomagnetic imaging applications.

## 1. Introduction

High-sensitivity atomic magnetometers (AMs), which detect the Larmor precession of optically pumped atoms, have been widely applied in areas such as space exploration [1,2,3,4], fundamental physics research [5,6,7,8], and biomagnetic measurements [9,10,11,12]. Biomedical magnetic sensing applications generally fall into two principal categories: the analysis of electric and magnetic properties of living systems closely related to their functionality (e.g., magnetocardiography (MCG) and magnetoencephalography (MEG)) and the analysis of specific properties of bioanalytes or magnetic label detection after their injection into living systems [13,14,15,16,17]. While technologies such as magnetoimpedance (MI) and magnetoresistive (MR) sensors have shown significant promise in detecting magnetic labels in blood vessels or intelligent measurement controls [13,16,17], spin-exchange relaxation-free (SERF) AMs are particularly distinguished by their ultrahigh sensitivity in detecting weak endogenous biomagnetic fields without the need for cryogenic cooling. In conventional optically pumped AMs, sensitivity is limited by the transverse relaxation rate induced by spin-exchange collisions among alkali–metal atoms. The SERF AM, pioneered by the Romalis group at Princeton University [18], eliminates this limitation by operating at high alkali–metal atomic densities and near-zero magnetic fields [19,20], where the spin-exchange collision rate greatly exceeds the Larmor precession frequency. With this mechanism, SERF AMs have achieved ultrahigh sensitivity, reaching 0.16 $\mathrm{fT}/{\mathrm{Hz}}^{1/2}$ in a gradiometer configuration [21], surpassing superconducting quantum interference devices (SQUIDs) and establishing themselves as the most sensitive magnetometers to date. In comparison with SQUIDs, SERF AMs provide non-cryogenic operation [22], ultrahigh sensitivity [21], adequate bandwidth [23,24], and promising prospects for wearable and cost-effective biomagnetic systems [9,25,26,27,28].

Many investigations have increasingly focused on the miniaturization and integration of SERF AMs. Two principal integration strategies have been developed: one incorporates the laser diode directly into the sensor head [29,30,31], whereas the other positions the laser externally with light delivered via polarization-maintaining optical fiber [32,33,34]. For the first approach, a typical example is the commercial development by QuSpin [29,35,36]. To mitigate the magnetic disturbances associated with laser temperature stabilization, a vertical-cavity surface-emitting laser chip is mounted on a non-magnetic heating plate for thermal control. While this strategy avoids the use of a thermoelectric cooler, it results in a relatively slow thermal response due to the absence of active cooling. Moreover, when the sensor head is fully integrated, heating of the vapor cell can further compromise the stability of the laser. In contrast, many research groups have adopted the second approach, particularly for multi-channel biomagnetic measurements. Building on the fiber-delivery approach, Kitching et al. leveraged Micro-Electro-Mechanical System (MEMS) technology to realize highly integrated sensor heads, combining alkali vapor cells, micro-optical components, heaters, and photodiodes into compact units of approximately 1 ${\mathrm{cm}}^{3}$ [32,37]. The combination of MEMS-based integration and optical fiber coupling has enabled the implementation of multi-channel SERF AM systems in which sharing a single laser among multiple sensor heads effectively suppresses common-mode noise originating from the light source [38]. Nevertheless, the focus on sensor-head miniaturization contrasts with the fact that other system components, including laser drivers, vapor-cell heating, magnetic-field coil control, and signal acquisition and processing, still largely depend on conventional laboratory instrumentation, which poses limitations for practical biomagnetic applications.

This article presents the development of a fiber-coupled fully integrated SERF AM featuring a compact sensor head designed in a single-beam configuration enabled by a polarization-maintaining fiber, together with a custom-designed instrument box. Here, “fully integrated” refers to the integration of both the sensor head and all the essential control and signal processing electronics, including the laser unit, vapor-cell temperature controller, magnetic field compensation and modulation circuits, and signal processing via a field-programmable gate array (FPGA) within a single self-contained instrument box. The incorporation of a closed-loop system ensures precise magnetic field control, maintaining sensor stability near zero and thereby enhancing the system bandwidth, which is a crucial factor in biomedical magnetic field measurements. In addition, the successful practical implementation of the fully integrated SERF AM in capturing both cardiac and alpha rhythm signals demonstrates its potential for seamless integration into MCG and MEG systems.

## 2. Experimental Setup

**Figure 1:**
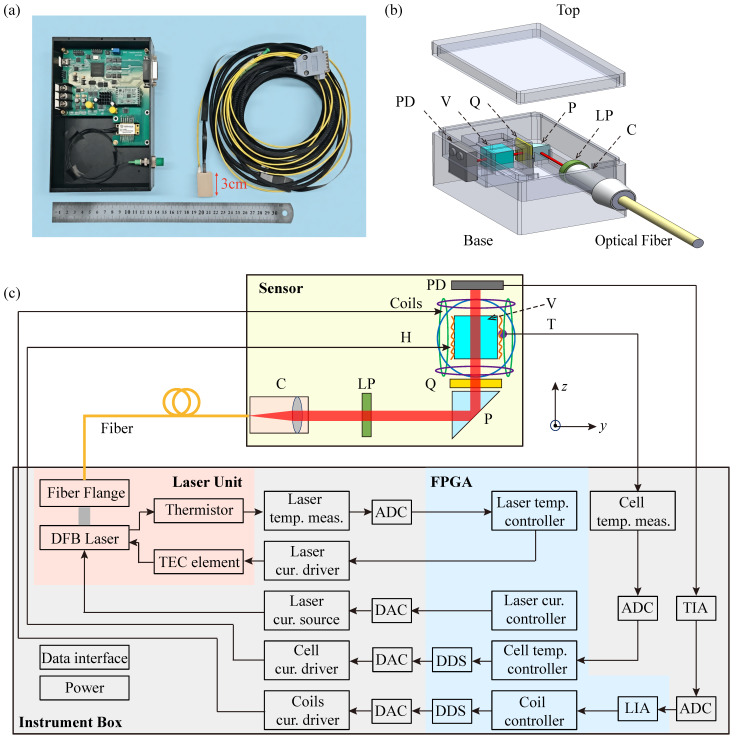
(**a**) Photograph of the fully integrated SERF AM, which consists of sensor module (right), instrument box (left), inbound polarization-maintaining optical fiber, and a cable. (**b**) Schematic diagram of the sensor module. (**c**) Schematic diagram of the optical path and electrical signal transmission arrangement inside SERF AM. C: fiber collimator; LP: linear polarizer; P: reflection prism; Q: quarter-wave plate; H: non-magnetic resistive heater; V: vapor cell; T: thermistor; PD: photodiode; TIA: transimpedance amplifier; ADC: analog-to-digital converter; LIA: lock-in amplifier; DAC: digital-to-analog converter; DDS: direct digital synthesis; FPGA: field-programmable gate array. Here, temp. and cur. are abbreviations for temperature and current, respectively.

Figure 1a shows a photograph of the fully integrated SERF AM sensor head together with its instrument box, which are connected via a polarization-maintaining fiber and electrical cable. The sensor module has compact outer dimensions of only 3.0 cm × 2.0 cm × 1.5 cm, and all its structural components are fabricated from the heat-resistant polyetheretherketone (PEEK). Our fully integrated SERF AM adopts a single-beam configuration for both pumping and probing the atomic polarization. The sensor module mainly comprises a fiber collimator, a vapor cell, a resistive heater, a photodiode, a set of coils, and associated optical components. A distributed feedback (DFB) laser (EYP-DFB-0795-00015-1500-BFY12-0005, Toptica Inc., Gräfelfing, Germany), operating at 795 nm and integrated into the instrument box, provides a linearly polarized beam that is coupled into the sensor module via a polarization-maintaining fiber connected to the collimator. The collimated beam, with a diameter of 1.0 mm, is converted to circular polarization by sequentially passing through a linear polarizer, a reflection prism, and a quarter-wave plate. After traversing the vapor cell, the transmitted beam is detected by a silicon photodiode, as illustrated in Figure 1b.

The vapor cell, serving as the sensitive element, contains a droplet of rubidium metal along with 760 Torr of nitrogen buffer gas. The nitrogen not only suppresses wall-collision-induced spin relaxation but also assists optical pumping by quenching the excited state [39,40]. Fabricated from borosilicate glass, the vapor cell has an outer dimension of 4 mm × 4 mm × 4 mm. Positioned approximately 6 mm from the cell center to the end of the sensor module, this compact configuration effectively mitigates signal attenuation in biomagnetic measurements.

The instrument box, built around an FPGA (XC6SLX25-2FTG256C, Xilinx Inc., CA, USA), integrates both the laser unit and the main control circuit board. The control board provides drive signals for the laser, magnetic field coils, and cell temperature controller while simultaneously processing the photodiode output, as shown in Figure 1c. Communication between the SERF AM and a personal computer is realized via a serial port interface, enabling transmission of the optical signal and control commands for parameters, such as laser current and temperature, vapor-cell temperature, and the strength of the compensation and modulation magnetic fields. To operate in the SERF regime, the vapor cell is heated to approximately 160 ℃, corresponding to a rubidium atomic density of $10^{14}$ cm^−3^. Non-magnetic heating tabs are mounted on the sides of the vapor cell and driven by a high-frequency alternating current at 244 kHz, generated via FPGA-based direct digital synthesis (DDS). Real-time temperature control is achieved using a non-magnetic Pt1000 thermometer (S233222PF, MINCO Inc., MN, USA), ensuring fluctuations remain below 15 mK at the operating point. A set of coils surrounding the vapor cell are employed both to compensate residual external magnetic fields and to provide a modulation field. To suppress compensation-field noise, the current is generated from a single 20-bit digital-to-analog converter (AD5791, Analog Devices Inc., MA, USA), providing a field compensation resolution better than one part per million of the full-scale range.

In SERF regime, the overall evolution of atomic polarization can be described by Bloch equation [41]. We assign the pump beam to propagate along the *z*-axis. After compensating the background magnetic field in the vapor cell, the magnetic fields along the *x*-axis and *z*-axis can be treated as negligible. When a modulation magnetic field of amplitude $B_{m}$ and frequency $w_{m}$ is applied along the *y*-axis, the atomic polarization is given by [42,43,44]

(1) $$P_{z}\left(w_{m}\right)=J_{0}\left(\frac{\gamma_{e}B_{m}}{qw_{m}}\right)J_{1}\left(\frac{\gamma_{e}B_{m}}{qw_{m}}\right)\frac{2R_{p}\gamma_{e}B_{0}}{{\left(R_{p}+R_{rel}\right)}^{2}+{\left(\gamma_{e}B_{0}\right)}^{2}}\cdot \sin\left(w_{m}t\right),$$

where $J_{0}$ and $J_{1}$ are the 0-order and 1-order Bessel functions of the first type, $\gamma_{e}$ is the electron gyromagnetic ratio, *q* is the nuclear slowing-down factor, $R_{p}$ is the rate of optical pumping, $R_{rel}$ is the spin relaxation rate, and $B_{0}$ is the magnetic field to be measured. Since $B_{0}$ to be measured is very small, the first harmonic of the transmitted light intensity is obtained by employing Taylor expansion at the equilibrium spin polarization [45].

In experiments, the transmitted light is detected by a silicon photodiode within the sensor module. The resulting current signal is then conveyed through a cable to a transimpedance amplifier (TIA) circuit located in the instrument box. Subsequently, the output voltage signal of TIA is digitalized by a low-noise analog-to-digital converter and then transmitted to the lock-in amplifier (LIA) in the FPGA. Within the LIA, a finite impulse response (FIR) filter is designed with a bandwidth of 155 Hz, well matched with the response of SERF AM while effectively suppressing noise. The phase accuracy of reference signal is designed to be 0.0044° so that it can maximize the signal to noise of measured magnetic field demodulated with LIA. Simultaneously, a LabVIEW software (version 2015) program is developed to implement the data acquisition, visualization, and control command transmission.

The proposed fiber-coupled fully integrated SERF AM represents a scalable and cost-effective solution for high-density biomagnetic sensing applications. The system combines a compact sensor head with a custom-designed instrument box. This instrument box integrates essential functionalities, including optimization of the laser unit, control of the vapor-cell heater, compensation and modulation of magnetic fields, and signal processing via an FPGA. These features ensure robust operation in the SERF regime. The architecture naturally supports multi-channel expansion. The DFB laser delivers a typical output power of 15 mW, which far exceeds the approximately 200 μW required per sensor head. A single laser can therefore serve multiple sensor heads through a fiber-optic splitter. Only the heater drivers, coil current sources, and signal acquisition circuits need to be replicated for each channel. This shared-laser approach reduces the per-channel hardware cost despite the high unit price of the DFB laser. It also enhances measurement consistency by ensuring identical common-mode optical noise across all channels. These characteristics make the system especially well suited for emerging applications, such as MCG and MEG. Both require large-scale arrays of high-sensitivity, low-cost, and spatially dense magnetic sensors.

## 3. Experimental Results and Discussion

### 3.1. SERF AM Characterization

**Figure 2:**
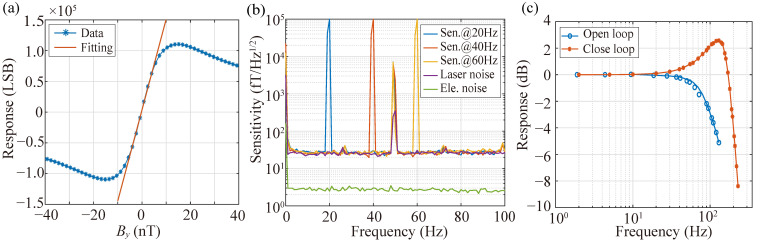
(**a**) Response signal with respect to the varying magnetic field. The blue and red traces are demodulated outputs of the LIA and the linear fitting result near a zero magnetic field, respectively. The unit of magnetometer output is Least Significant Bit (LSB). (**b**) Magnetic sensitivity and noise spectrum. The blue, red, and yellow traces are the magnetic sensitivity under modulation fields at 20 Hz, 40 Hz, and 60 Hz. The purple trace is laser noise, and the green trace is electronic noise. (**c**) The normalized amplitude–frequency responses in open-loop (blue trace) and closed-loop (red trace) modes, respectively.

To characterize the performance of the SERF AM, measurements are carried out inside a five-layer μ-metal magnetic shield, where the residual magnetic field is suppressed below 1 nT. A large solenoid coil (24 nT/mA), calibrated with a commercial fluxgate magnetometer (CTM-6W, National Institute of Metrology, Beijing, China), provides a calibration field along the *y*-axis, driven by a function generator (33600A, KEYSIGHT Inc., AZ, USA). Prior to sensitivity and bandwidth characterization, the background magnetic field around the vapor cell is compensated to near zero using the internal coil set. By sweeping the solenoid-generated field from negative to positive values, a zero-field resonance signal is observed. This signal, obtained through photodiode detection of transmitted laser light and subsequent amplification by the TIA, is further analyzed by applying a 976 Hz modulation field. The dispersive resonance line shape is then extracted and synchronously demodulated using an FPGA-based LIA, which effectively suppresses technical 1/f noise. Figure 2a shows the dispersive resonance signal together with the fitted curve around zero field, yielding a scale factor of 14775 LSB/nT.

Figure 2b depicts the magnetic sensitivity of the SERF AM in the open-loop mode, derived by recording the output for 100 s and analyzing the power spectral density under sinusoidal calibration fields at amplitudes of 100 pTrms and frequencies of 20 Hz, 40 Hz, and 60 Hz, respectively. To acquire a more accurate sensitivity, the power spectral density is averaged within every 1 Hz range. The sensitivity of the SERF AM is 25 $\mathrm{fT}/{\mathrm{Hz}}^{1/2}$@5∼100 Hz, except for some technological noise. The green and purple lines represent the electronic noise and laser noise, respectively. Electronic noise is assessed by computing the power spectral density of the magnetometer output and dividing the scalar factor when the DFB laser is deactivated. From Figure 2b, the electronic noise is approximately 2.8 $\mathrm{fT}/{\mathrm{Hz}}^{1/2}$ over 2∼100 Hz. Given that the electronic noise is significantly lower than the magnetic noise in the SERF AM, we can utilize the in-phase output of the LIA, which is insensitive to magnetic fields, in a similar manner as the laser noise. The determined laser noise is about 24 $\mathrm{fT}/{\mathrm{Hz}}^{1/2}$ over 2∼100 Hz. This noise primarily stems from the instability observed in laser power and frequency, significantly contributing to limiting the sensitivity of the system. Meanwhile, the comparison between magnetic field sensitivity and laser noise also indicates that the total magnetic noises, mainly including the Johnson noise of the five-layer magnetic shield and the magnetic noise of the compensating coil, are less than 7 $\mathrm{fT}/{\mathrm{Hz}}^{1/2}$.

In addition, to evaluate the frequency response, we record the amplitude of the SERF AM output under the sinusoidal calibration fields of 100 pTrms at various frequencies. Figure 2c shows the normalized amplitude frequency responses in both open-loop and closed-loop modes. In open-loop mode, the lock-in amplifier output is directly used as the magnetic field measurement, yielding a linear response only in the vicinity of the zero-field point. In closed-loop mode, the same output serves as an error signal for an FPGA-implemented Proportional–Integral (PI) controller that drives compensation coils to maintain the atomic ensemble at zero field, and the calibrated coil drive current then serves as the linear field measurement output. The −3 dB bandwidth of the SERF AM in open loop is approximately 100 Hz. However, accommodating higher frequency signals in MEG measurements requires expanding the bandwidth, typically achieved by enhancing transverse spin relaxation. It is crucial to note that this extension significantly impacts the sensitivity of the SERF AM. By employing the closed-loop system, which maintains the magnetic field close to zero at the sensor, the bandwidth is extended to 193 Hz. The resonance peak observed in the closed-loop response is an inherent characteristic of second-order control systems. As the bandwidth expands, the system’s damping ratio decreases, causing energy accumulation at a specific frequency and producing the observed peak. This phenomenon reflects the fundamental trade-off between bandwidth extension and system stability rather than a design flaw. The PI feedback parameters govern the closed-loop bandwidth, with higher gains increasing bandwidth at the expense of sensitivity, while lower gains preserve sensitivity but reduce the response speed. Therefore, PI tuning requires a balance between bandwidth and sensitivity.

### 3.2. MCG Measurement

The MCG signal originates from the electrical activity of the heart, driven by the rhythmic contraction and relaxation of its muscles throughout each heartbeat. This changing electrical activity in the heart generates a weak but detectable magnetic field around the body. It offers insights into cardiac rhythm, timing, and potential irregularities, aiding in the diagnosis and monitoring of various heart conditions. Additionally, MCG contributes to advancing our understanding of cardiac physiology and pathophysiology, facilitating the development of improved diagnostic and therapeutic strategies for heart-related disorders.

**Figure 3:**
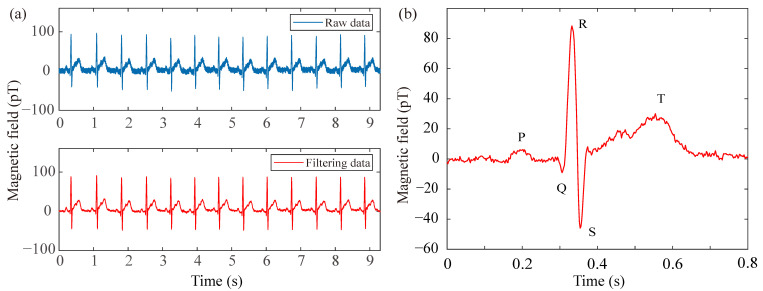
MCG signal measurement by the fully integrated SERF AM. (**a**) The time-domain MCG signal including both raw and filtered data. (**b**) Real-time filtered unaveraged MCG signal (one heartbeat cycle).

In the pursuit of recording typical MCG signals with the fully integrated SERF AM in the open-loop mode, a healthy adult volunteer (34 years old and one of the authors) is positioned inside a magnetic shield to minimize ambient interference. The sensor is securely attached to the chest of the subject to optimize the signal-to-noise ratio. Stable breathing is maintained throughout the experiment to mitigate measurement uncertainty associated with sensor-position fluctuations. To address power-line interference at 50 Hz, the raw data are processed with a digital notch filter, thereby enhancing the visibility of the cardiac signal. Figure 3a presents both the raw and filtered MCG traces over a 9.3 s interval, demonstrating a clear improvement after filtering. Furthermore, Figure 3b displays the real-time unaveraged filtered MCG waveform corresponding to a single heartbeat cycle. Distinctive features, including the QRS complex as well as the P and T waves, are clearly resolved. Although simultaneous electrocardiograph (ECG) was not recorded in this specific sensor-validation experiment, the observed waveform morphology and temporal characteristics align with standard physiological norms established in the clinical MCG literature [14,15]. Specifically, the QRS complex duration and the relative timing of the P and T waves correspond to expected cardiac cycle intervals, confirming the physiological origin of the detected magnetic signal. The continuous 9.3 s recording inherently enables continuous heart rate monitoring through standard R-peak detection algorithms. By extracting consecutive R-R intervals from the time-series data, instantaneous heart rate (HR = 60/R-R) can be computed continuously without additional measurements. This capability is fundamentally supported by the SERF AM’s bandwidth, which substantially exceeds the spectral requirements for heart rate variability and preserves the morphological features of individual cardiac cycles. These results demonstrate not only the feasibility of employing the fully integrated SERF AM for practical MCG measurement but also its potential utility in future biomagnetic applications.

### 3.3. MEG Measurement

To further evaluate the performance of the fully integrated SERF AM, we also carry out a human experiment involving photic blocking response. Photic blocking, such as closing eyes, typically induces an alpha rhythm signal primarily originating from the occipital region, displaying frequencies ranging from 8 Hz to 13 Hz [46]. The alpha rhythm tends to increase when the eyes are closed and decrease when they are open, making it a notable marker in studying states of consciousness, cognitive processes, and sensory inhibition. On the basis of these characteristics, we utilize the SERF AM in the open-loop mode to capture and analyze the alpha rhythm MEG signal.

**Figure 4:**
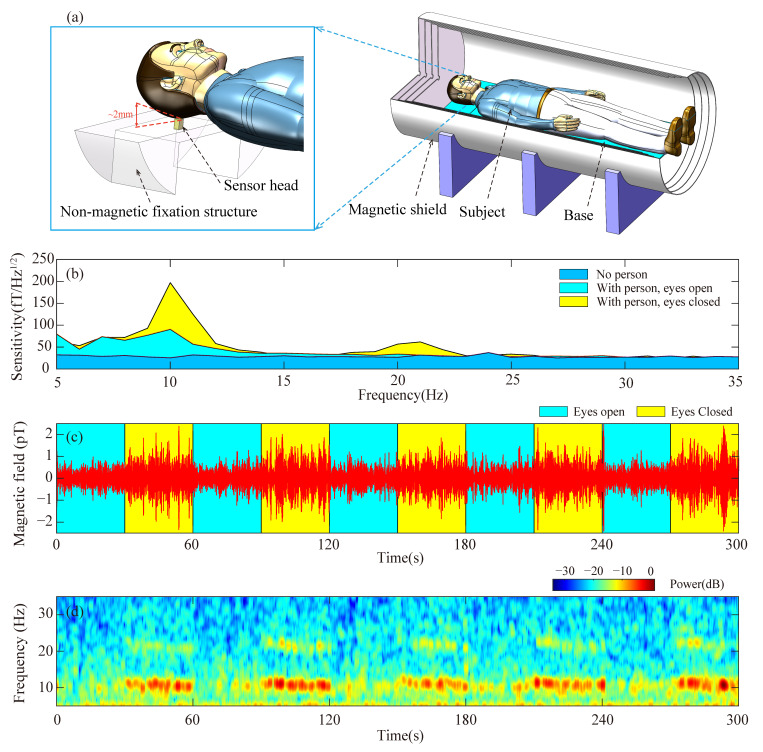
Alpha rhythm MEG signal measurement. (**a**) Schematic diagram of the MEG experimental setup inside the magnetic shield, depicting the sensor head, subject’s head, and non-magnetic fixation structure with mechanical stabilization for vibration suppression. (**b**) Displays the power spectral density between 5 Hz and 35 Hz in the absence of a person (blue region) while showcasing the measured results when an individual closes (yellow) and opens (cyan) his eyes. (**c**) Exhibits the time-domain representation of the alpha rhythm signal, with the yellow and cyan regions denoting the eyes-closed and eyes-open states, respectively. (**d**) Presents the short-time Fourier transform spectrum derived from the measured alpha rhythm signal.

To ensure experimental stability and effectively mitigate vibration and motion artifacts, the sensor head is rigidly secured to a custom non-magnetic fixation structure positioned within the magnetic shield, as schematically depicted in Figure 4a. Subsequently, a healthy adult (aged 34, one of the authors) lies inside the magnetic shield, aligning his occipital region as closely as possible to the sensor. The sensor head surface is positioned approximately 2 mm from the subject’s scalp, as illustrated in Figure 4a. Given the internal design where the vapor-cell center is located 6 mm from the sensor surface, this configuration ensures close proximity to the neural source. This stand-off distance is mechanically maintained by the fixation structure to suppress vibration-induced noise. For comparative analysis, the subject maintains closed-eyes and open-eyes conditions for 100 s each. In Figure 4b, the blue region shows the power spectral density within the 5 Hz to 35 Hz frequency range in the absence of an individual. The yellow and cyan regions represent the recorded spectrum when the subject closes and opens his eyes, respectively. Figure 4b distinctly displays the alpha rhythm signal around 10 Hz when the subject’s eyes are closed, yet the signal experiences attenuation upon eyes opening. Additionally, a minor peak near 20 Hz is evident in the yellow region, which may be associated with the second harmonic of the alpha rhythm signal [47], although further validation with simultaneous electroencephalograph (EEG) is required to confirm this origin.

To validate the signal’s viability, the subject is instructed to cyclically alternate between opening and closing their eyes every 30 s. The resulting alpha rhythm signal, obtained through a bandpass filter ranging from 7 Hz to 14 Hz, is depicted in Figure 4c. The yellow and cyan regions correspond to the eyes-closed and eyes-open states, respectively. A noticeable discrepancy in signal amplitude is evident between these two states. Additionally, Figure 4d illustrates the short-time Fourier transform spectrum derived from the measured alpha rhythm signal presented in Figure 4b. Detection of the alpha rhythm provides only a foundational validation of the neural signal acquisition capability of our SERF AM. It should be noted that alpha rhythm testing alone is insufficient for comprehensive clinical MEG validation. More rigorous validation approaches, such as evoked potential measurements or simultaneous EEG comparison, will constitute essential follow-up work for advancing the system toward practical MEG applications.

### 3.4. Discussion

The 25 $\mathrm{fT}/{\mathrm{Hz}}^{1/2}$ sensitivity and 193 Hz closed-loop bandwidth achieved by our fully integrated SERF AM represent typical to superior performance among miniaturized systems designed for practical biomagnetic applications. Laboratory-scale SERF systems have demonstrated record sensitivities approaching 0.16 $\mathrm{fT}/{\mathrm{Hz}}^{1/2}$ in gradiometer mode [21], and such architectures remain incompatible with dense multi-channel arrays owing to their substantial size, operational complexity, and dependence on auxiliary instrumentation. Commercial integrated-laser sensors such as QuSpin achieve sensitivity of approximately 15 $\mathrm{fT}/{\mathrm{Hz}}^{1/2}$ with bandwidth of 100 Hz [29,35,36] but embed the laser diode directly within each sensor head, which limits scalability for large arrays and introduces thermal crosstalk between the laser and vapor cell.

It is important to contextualize these performance metrics against other magnetic sensing modalities used in biomedical applications. For instance, MI and MR sensors offer compact form factors and have been successfully utilized for detecting magnetic labels in biological tissues and mimicking implants [13,16,17]. Recent work by Yaga et al. demonstrated MCG recording using magnetoresistive sensors outside a shielded room, highlighting the robustness of MR technology in noisy environments [14]. However, these sensors typically operate in the picotesla to nanotesla range or require specific magnetic labeling, whereas our SERF AM achieves femtotesla sensitivity that is suitable for unlabeled endogenous neural and cardiac field detection. Furthermore, while clinical MCG is moving towards unshielded configurations [15], the ultrahigh sensitivity of SERF AMs provides a distinct advantage for source localization accuracy in multi-channel arrays where signal amplitude is critical.

In contrast, our system delivers a balanced performance envelope: the 25 $\mathrm{fT}/{\mathrm{Hz}}^{1/2}$ sensitivity fully satisfies the requirements for biomagnetic measurements, while the 193 Hz bandwidth exceeds the dynamic range needed for cardiac signals and neural oscillations. This dual adequacy is experimentally validated by our successful recording of unaveraged P, QRS, and T waves together with alpha rhythm signals, confirming that both sensitivity and bandwidth meet the demands of practical biomagnetic signal detection.

Regarding cost effectiveness for scalable deployment, our fiber-coupled architecture provides a decisive advantage through laser sharing across multiple sensor channels. A single 15 mW DFB laser integrated in the custom-designed instrument box theoretically possesses the power budget to serve up to 70 sensor heads, each requiring approximately 200 μW of optical power. We acknowledge that practical channel counts will be lower due to fiber-splitting insertion losses, coupling inefficiencies, and power uniformity requirements. However, even with a conservative splitting ratio such as 1:32, the shared-laser architecture still offers significant cost and consistency advantages over independent laser sources per channel. This architecture eliminates the need to replicate expensive laser components across channels as only heater drivers, coil current sources, and signal acquisition circuits require per-channel replication, all of which possess substantially lower unit costs than laser diodes. The shared-laser approach not only reduces hardware expenses but also enhances measurement consistency by ensuring identical optical common-mode noise across all channels, thereby facilitating effective gradiometric noise cancellation. For biomagnetic imaging systems requiring approximately 36 channels for MCG and 275 channels for whole-head MEG, this architecture offers superior scalability and cost efficiency compared with one-laser-per-sensor designs as the modular design allows MCG arrays to be accommodated by a single unit and enables scalable expansion of MEG systems through multiple shared-laser units.

## 4. Conclusions

In summary, this study presents the development of a fully integrated SERF AM based on a single-beam configuration consisting primarily of a sensor module and an instrument box. The sensor module, with a volume of 9 ${\mathrm{cm}}^{3}$, incorporates a collimator, vapor cell, resistive heater, photodiode, a set of coils, and various other optical components. Conversely, the corresponding control electronics, such as the laser controller, cell temperature controller, and coil driver, are implemented via an FPGA. Employing a feedback control system to maintain the magnetic field at the sensor close to zero significantly enhances the bandwidth by almost twofold, meeting the necessary criteria for biomagnetic measurements. Notably, we achieve a sensitivity of 25 $\mathrm{fT}/{\mathrm{Hz}}^{1/2}$@5∼100 Hz with a −3 dB bandwidth of 193 Hz. Furthermore, experiments involving adult MCG and alpha rhythm MEG signals conducted using the fully integrated SERF AM yield promising outcomes. The distinct features of the MCG signal, including the QRS complex, P, and T waves, are accurately identified. Additionally, successful observation of the alpha rhythm signal associated with closed eyes is achieved. The demonstrated high performance of the fully integrated SERF AM positions it as a highly promising technology for forthcoming MEG and MCG applications in the near future.

## Data Availability

The data that support the findings of this study are available from the corresponding author upon reasonable request.
